# Simultaneous bilateral inflammatory choroidal neovascularization in a case of healed serpiginous-like choroiditis

**DOI:** 10.3205/oc000199

**Published:** 2022-05-20

**Authors:** Gitanjli Sood, Ramanuj Samanta, Devesh Kumawat, Prateek Nishant

**Affiliations:** 1 Department of Ophthalmology, All India Institute of Medical Sciences (AIIMS), Rishikesh, Uttarakhand, India; 2 Department of Ophthalmology, Lady Hardinge Medical College, New Delhi, India; 3 Department of Ophthalmology, All India Institute of Medical Sciences (AIIMS), Patna, Bihar, India

**Keywords:** inflammatory choroidal neovascularization, serpiginous-like choroiditis, anti-VEGF, posterior uveitis, TB choroiditis

## Abstract

**Objective:**

Inflammatory choroidal neovascularization (i-CNV) is an infrequent but sight-threatening complication of posterior uveitis. Although it can occur in a wide range of infectious and non-infectious uveitides, presence of simultaneous bilateral i-CNV is rare. In this report, we present a unique case of bilateral simultaneous i-CNV in a young patient of healed tubercular serpiginous-like choroiditis.

**Method:**

A 20-year-old male presented with recent worsening of vision in the right eye for one month. Fundus examination revealed bilateral multifocal healed choroiditis lesions with right eye tiny subfoveal hemorrhage raising the suspicion of an underlying choroidal neovascularization. Fundus fluorescein angiography and optical coherence tomography confirmed presence of choroidal neovascular membrane in both eyes.

**Result:**

Resolution of activity was noted in both eyes after bilateral sequential intravitreal bevacizumab injections.

**Conclusion:**

Inflammatory choroidal neovascularization may be seen in patients with healed tubercular serpiginous-like choroiditis, after a long period of quiescence. Simultaneous bilateral presentation is rare but possible, requiring mandatory multimodal imaging of both eyes under high index of suspicion. Early institution of anti-vascular endothelial growth factor may salvage optimum vision in such a scenario.

## Introduction

Inflammatory choroidal neovascularization (i-CNV) is an uncommon complication of posterior uveitis, rarer with bilateral disease [1]. Diagnosis is often difficult in presence of pre-existing scarring, simultaneous choroiditis lesions, cataract and vitreous haze [2]. Ancillary investigations like optical coherence tomography (OCT) and fundus fluorescein angiography (FFA) may help in diagnosis of the clinically doubtful cases [3]. It can lead to permanent drop in vision if missed and not treated on time [4]. This case highlights the diagnosis of i-CNV in one eye based on patient’s symptom and clinical cue; i-CNV in the fellow eye was confirmed on FFA in the pretext of equivocal fundus findings.

## Case description

**Figure 1:**
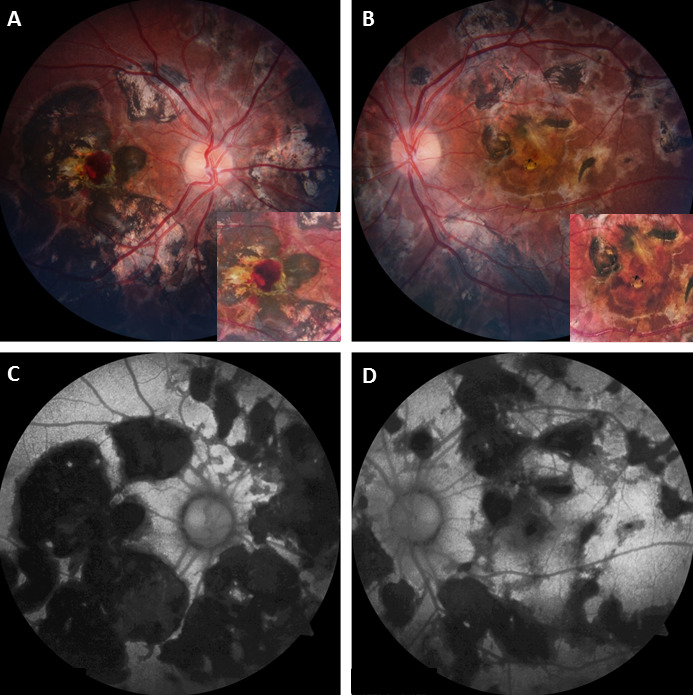
Fundus photo of the right eye (A) and the left eye (B) at presentation showing multiple pigmented healed choroiditis patches of variable size at the posterior pole. A small retinal haemorrhage adjacent to yellowish membranous lesion (inset; A) and a suspicious yellowish membranous lesion (inset; B) was also noted. Fundus autofluorescence of the right eye (C) and the left eye (D) at presentation showing uniform hypoautofluorescence over the choroiditis lesions suggestive of healed lesions.

**Figure 2:**
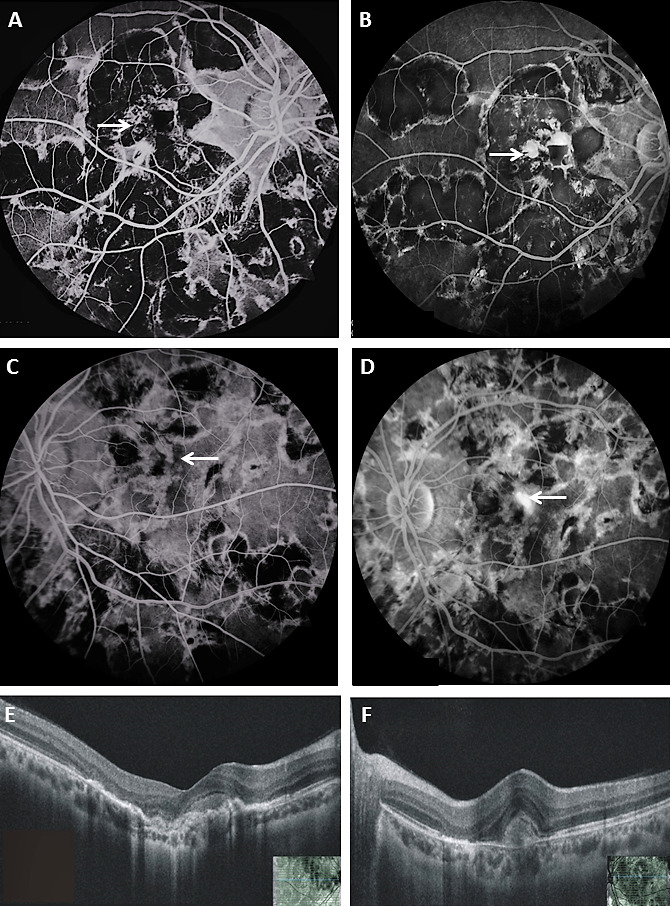
FFA of the right eye in arterio-venous phase (A) showing hypofluorescent patches with hyperfluorescent borders and an area of blocked fluorescence at the fovea with surrounding punctate fluorescence (white arrow) that increases and leaks in late phase (B) suggestive of CNVM. FFA of the left eye (C–D) also reveals a juxta-foveal area of hyperfluorescence (white arrow) compatible with CNVM. OCT line scan through the fovea at presentation shows a subfoveal hyperreflective membrane with overlying trace fluid and mild retinal thickening in the right eye (E) and the left eye (F).

A 20-year-old Asian-Indian male presented with bilateral painless diminution of vision for five years with recent worsening in the right eye (OD) for last one month. Ocular examination revealed best-corrected visual acuity (BCVA) of 20/200 in OD and 20/120 in the left eye (OS). Intraocular pressures and anterior segments were unremarkable. Dilated fundus showed (Figure 1A–B (Fig. 1)) bilateral multiple well-defined choroiditis lesions with overlying pigment clumping at the posterior pole and mid-periphery. There was no associated vitritis. Careful examination also revealed a small subretinal hemorrhage close to the fovea in OD (inset; Figure 1A (Fig. 1)) and a doubtful yellowish subretinal lesion adjacent to the central pigmented healed choroiditis scar in OS (inset; Figure 1B (Fig. 1)). Fundus autofluorescence (FAF) of both eyes (Figure 1C–D (Fig. 1)) showed uniform hypoautofluorescence over choroiditis patches suggestive of healed lesions. FFA (Figure 2A–D (Fig. 2)) and OCT (Figure 2E–F (Fig. 2)) was suggestive of bilateral i-CNV.

Review of records revealed an induration of 15×10 mm on tuberculin skin test, non-reactive VDRL, normal chest imaging and unremarkable laboratory reports. Past medical history revealed a diagnosis of tubercular meningitis with associated hydrocephalus at 10-years of age. The patient received antitubercular therapy (ATT) for nine months during that episode and also underwent ventriculoperitoneal shunt surgery.

Based on the clinical findings and ancillary investigations, a diagnosis of bilateral i-CNV associated with healed tubercular serpiginous-like choroiditis (SLC) was made. The patient underwent bilateral intravitreal bevacizumab (IVB; 1.25mg/0.05ml) injection at two weeks interval (OD treated first). After a month, OD injection was repeated due to persistent i-CNV.

**Figure 3:**
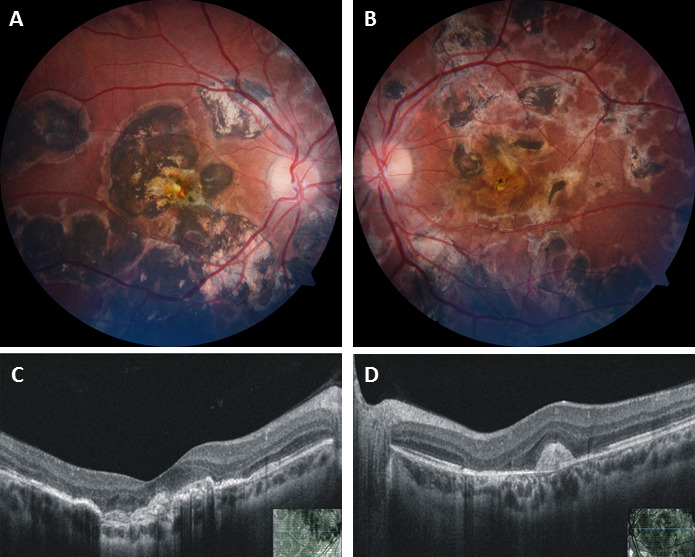
Follow-up fundus photo of the right eye (A) and the left eye (B) at six months showing near total resolution of hemorrhage in the right eye and subfoveal scar in both eyes. OCT at final follow-up after six months in the right eye (C) and the left eye (D) reveals an organized subfoveal scar in both eyes without any surrounding edema or fluid.

The patient was followed up monthly thereafter and at six-months follow-up, his BCVA remained stable at 20/120 in both eyes. Fundus showed resolution of i-CNV (Figure 3A–B (Fig. 3)) and OCT showed bilateral subfoveal organized membrane without any associated intra-retinal/sub-retinal fluid (Figure 3C–D (Fig. 3)). The patient was advised monthly follow-up to look for any recurrence.

## Discussion

I-CNV is an uncommon but potentially sight-threatening complication, mostly associated with posterior uveitis or panuveitis (2–2.7%), less commonly with anterior uveitis and rarely with intermediate uveitis [1]. Eyes with i-CNV in one eye have a seven-fold increased risk of developing it in the fellow eye. Various infectious (histoplasmosis, toxoplasmosis, toxocariasis, tuberculosis) and non-infectious (Vogt-Koyanagi-Harada’s disease, punctate inner choroidopathy, multifocal choroiditis, serpiginous choroiditis) uveitis have been reported to be associated with i-CNV [1], [3]. However, exact prevalence of i-CNV following intraocular tuberculosis is unknown as it is uncommon [3]. In a retrospective study from the Indian subcontinent involving 49 eyes with i-CNV, tubercular etiology was noted in 14 eyes. Among these, only two eyes had SLC [5].

While extrafoveal i-CNV is mostly asymptomatic and incidentally detected on imaging, lesions close to the fovea may present with diminution of vision or new onset metamorphopsia. Diagnosis of such lesions are challenging and are often missed due to various factors like significant background scarring, intense pigmentation, poorly dilating pupil, cataract, vitreous haze and sunset glow of fundus [2], [3]. A small peripapillary lesion or tiny subretinal hemorrhage are frequently overlooked until more obvious fundus changes occur or the patient presents with symptoms. In our case also, the patient was seen at multiple clinics elsewhere before presenting to us and was advised conservative management in view of healed lesions. A small subretinal hemorrhage in OD and a suspicious yellowish lesion in OS prompted us to investigate thoroughly. Although OD had a visible diagnostic clue, diagnosis in OS could have been missed unless the patient was subjected to FFA and OCT.

The lesions of i-CNV need to be differentiated from active choroiditis patches because treatment differs. Both can present clinically as yellowish lesions at the posterior pole with ill-defined margins with or without overlying fluid. On OCT, i-CNV presents mostly as type 2 lesion; another distinctive feature is the ‘pitchfork sign’ in which there are hyperreflective projections from the membrane to the overlying retina [6]. Active choroiditis lesions may show disruption in the outer retina with edema in OCT, but there is deeper penetration of OCT signal beneath the lesion, which is absent in CNVM [7].

The majority of i-CNV present as classic lesion in FFA; focal breach in retinal pigment epithelium (RPE) due to infection and inflammation is responsible for growth and progression of the neovascular membrane in the outer retinal space [3], [8]. In the early phase of FFA, the CNVM is iso- or hyperfluorescent, while active lesions are hypo- or isofluorescent. Late leakage is present in both, and presence of extensive scarring can make interpretation difficult. FFA may be more sensitive in detecting CNVM in patients with multifocal choroiditis than OCT [8], especially in those without substantial fluid or cystic spaces on OCT. Thus, even in absence of fluid on OCT, suspicious cases should undergo FFA to rule out active CNVM, as shown in our case also. Indocyanine green angiography (ICG) can also provide additional information regarding the primary choroidal pathology, which might have contributed to the formation of i-CNV.

OCT-angiography (OCTA) may be the only investigative modality that helps us differentiate between active choroiditis and i-CNV with certainity, especially in equivocal cases. OCTA helps in identifying the vascular network even in cases with inconclusive FFA [9], [10]. There will be no abnormal blood flow signals over an inflammatory lesion [11]. Up to 14% additional lesions were picked up on OCTA that were missed on conventional FFA and OCT [10], [12]. Although OCTA could not be performed in this case, i-CNV was confirmed using other imaging modalities.

Inflammation and angiogenesis both need to be managed in a case of i-CNV [13]. As compared to age-related macular degeneration (ARMD), these patients require fewer anti-VEGF injections (average 2.7) with lesser recurrences due to well-defined focal nature of the membrane [3], [9]. Associated active inflammation may need treatment with steroids and/or immunosuppressive medications to get the best outcome and reduce recurrences. Laser photocoagulation and photodynamic therapy were also used in the past, but anti-VEGF agents are the accepted primary treatment of i-CNV in the current era [14]. Roy et al., in their study including thirty eyes of i-CNV of varied etiology, have demonstrated improvement of visual acuity in 53.3% and stabilization in 26.6% eyes, with mean 2.76 anti-VEGF injections in a mean follow-up duration of 17.93±14.28 months [13]. In another study by Lott et al. including 34 i-CNV eyes, improvement of visual acuity was noted in 17% and stabilization in 33% at six months follow-up with a mean of two IVB injections [15]. Kim et al. have reported four cases of CNVM associated with presumed tubercular chorioretinitis and demonstrated favourable outcome with ATT, oral corticosteroids, and intravitreal anti-VEGF agents [16].

Occurrence of i-CNV following tubercular SLC is infrequently reported in literature despite tuberculosis being one of the leading causes of infectious uveitis in endemic regions like the Indian subcontinent. Invernizzi et al. have described five cases of i-CNV as a rare and unusual presenting sign of intraocular tuberculosis from a non-endemic country, which were initially misdiagnosed as exudative ARMD [17].

## Conclusion

This report is unique in depicting simultaneous bilateral presentation of i-CNV following healed tubercular SLC and emphasizes the importance of detailed examination of the fellow eye with appropriate ancillary imaging under high index of suspicion. Early treatment with anti-VEGF can be promising, although they may require long-term follow-up to look for any recurrences.

## Notes

### Competing interests

The authors declare that they have no competing interests.
